# Arteriovenous Fistula Caused by Ruptured Abdominal Aortoiliac Aneurysm

**DOI:** 10.5334/jbsr.3695

**Published:** 2024-08-19

**Authors:** Leizhi Ku, Yuhang Wang, Xiaojing Ma

**Affiliations:** 1 Department of Radiology, Wuhan Asia Heart Hospital Affiliated Wuhan University of Science and Technology, No.753 Jinghan Road, Hankou District, Wuhan 430022, P.R. China; 2 Department of Radiology, Wuhan Asia Heart Hospital Affiliated Wuhan University of Science and Technology, No.753 Jinghan Road, Hankou District, Wuhan 430022, P.R. China; 3 Department of Echocardiography, Wuhan Asia Heart Hospital Affiliated Wuhan University of Science and Technology, Wuhan 430022, P.R. China

**Keywords:** Aorto-iliac aneurysms, Ilio-iliac arteriovenous fistula, computed tomography angiography, endovascular repair

## Abstract

*Teaching point:* A ruptured aorto-iliac aneurysm, complicated by an iliac arteriovenous fistula, is rare but has a possibly fatal outcome and requires prompt diagnosis and appropriate treatment.

## Case History

**Figure 1:**
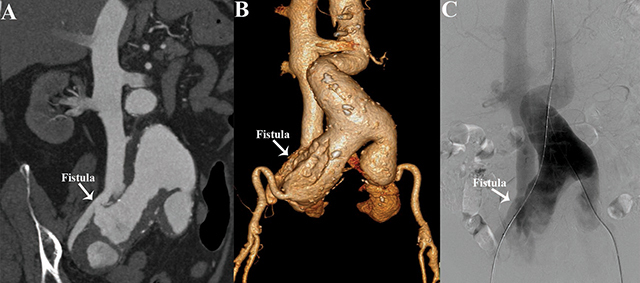
(**A** and **B**) Multiplanar reconstructions (MPR) and 3D volume rendered (3D-VR) CTA images show a large ruptured abdominal aortoiliac aneurysm causing an ilio-iliac arteriovenous fistula. (**C**) Abdominal aortography Abdominal aortography reveals ilio-iliac AVF with a fistula size of 8.5mm.

A 74–year-old female presented with an 8-hour history of sudden-onset lower abdominal pain. The physical examination revealed a blood pressure of 96/52 mmHg. Her symptoms were significantly relieved after she received analgesic therapy and cardiovascular support. A contrast-enhanced computed tomography angiography (CTA) examination was performed. Maximum-intensity projection CTA showed an infrarenal abdominal aortic aneurysm extending into the common iliac artery. The right common iliac artery aneurysm ruptured into the right common iliac vein, with the early appearance of contrast medium in the dilated inferior vena cava, indicative of the presence of an arteriovenous fistula (AVF) (Figure 1A). Three-dimensional volume-rendering CTA identified the aneurysms and the fistula orifice (Figure 1B). Abdominal aortography confirmed that the fistula was located between the right common iliac artery aneurysm and the common iliac vein (Figure 1C and Video S1). The patient underwent an emergency endovascular stent-graft repair.

## Comment

A spontaneous ilio-iliac arteriovenous fistula (AVF) caused by a ruptured abdominal aorto-iliac aneurysm is a rare condition. Patients with this condition may present with manifestations of high-output cardiac failure, vague abdominal pain, hematuria, lower extremity swelling, or multi-organ failure. Early diagnosis enables significant clinical improvement due to the early reversal of multi-organ complications. CTA is thus a key imaging modality to identify the site of the fistula, especially when clinical symptoms are not typical, and provides a road map for endovascular management [1]. Surgical mortality of an AVF is high because of hemorrhagic shock, high-output heart failure, renal failure, and massive blood loss during the operation. Endovascular repair is a less invasive alternative, with less hemorrhagic complications and limited hemodynamic deterioration during the intervention. Several reports show good early results of endoluminal repair of an AVF.

## Additional File

The additional file for this article can be found as follows:
